# Oral Dysbiotic Communities and Their Implications in Systemic Diseases

**DOI:** 10.3390/dj6020010

**Published:** 2018-04-16

**Authors:** Preethi Sudhakara, Abishek Gupta, Anshumouli Bhardwaj, Aruni Wilson

**Affiliations:** 1 Department of Genetic Engineering, SRM University, Chennai 603203, India; miyupreethi@gmail.com (P.S.); abhi.dbs2009@gmail.com (A.G.); 2 Regional Center for Biotechnology, New Delhi 110029, India; biotechbhardwaj@gmail.com; 3 Division of Microbiology and Molecular Genetics, School of Medicine, Loma Linda University, Loma Linda, CA 92350, USA; 4 Musculoskeletal Diseases Center, VA Loma Linda, Department of Veterans Affairs, Loma Linda, CA 92350, USA

**Keywords:** oral dysbiosis, human oral microbiome, yet-un cultivable organisms, systemic diseases

## Abstract

The human body supports the growth of a wide array of microbial communities in various niches such as the oral cavity, gastro-intestinal and urogenital tracts, and on the surface of the skin. These host associated microbial communities include yet-un-cultivable bacteria and are influenced by various factors. Together, these communities of bacteria are referred to as the human microbiome. Human oral microbiome consists of both symbionts and pathobionts. Deviation from symbiosis among the bacterial community leads to “dysbiosis”, a state of community disturbance. Dysbiosis occurs due to many confounding factors that predispose a shift in the composition and relative abundance of microbial communities. Dysbiotic communities have been a major cause for many microbiome related systemic infections. Such dysbiosis is directed by certain important pathogens called the “keystone pathogens”, which can modulate community microbiome variations. One such persistent infection is oral infection, mainly periodontitis, where a wide array of causal organisms have been implied to systemic infections such as cardio vascular disease, diabetes mellitus, rheumatoid arthritis, and Alzheimer’s disease. The keystone pathogens co-occur with many yet-cultivable bacteria and their interactions lead to dysbiosis. This has been the focus of recent research. While immune evasion is one of the major modes that leads to dysbiosis, new processes and new virulence factors of bacteria have been shown to be involved in this important process that determines a disease or health state. This review focuses on such dysbiotic communities, their interactions, and their virulence factors that predispose the host to other systemic implications.

## 1. Introduction

Human microbiome plays a pivotal role in human biology through its influence on many physiological functions such as human development, physiology, immunity, and nutrition. Even though the composition of the human microbiome has received considerable attention in recent years, the precise mechanisms whereby these microbial communities mediate disease and restore and maintain health remain unexplored. However, recent studies have shown that several chronic diseases of the mouth and gastro-intestinal tract are associated with alterations in the composition of the microbiome termed as “dysbiosis”. Dysbiosis is a significant harmful shift in the relative abundances and individual components of the microbiome which varies with their composition and relative abundances during health status. This shift causes major dysbiosis related diseases in humans, namely, periodontitis, irritable bowel syndrome, chronic vaginosis, etc. Among them, periodontal disease depicts a major dysbiotic condition due to the diversity of genera involved in normal and periodontal microbiome. Oral microbiota consists of two major types of bacteria: Gram-positive and Gram-negative bacteria with more than 700 species of microorganisms found in the oral cavity [1].

The etiology of periodontitis with both Gram-positive and Gram-negative bacteria suggests a complex heterogeneous microbial population which could be classified as early and late colonizers [2]. The oral cavity also includes several discrete microbial habitats such as gingival sulcus, teeth, attached gingiva tongue, lip, cheek, hard and soft palate [3]. With a steady transition of various environments such as the oxygen tension and nutrient availability, the bacterial microbiota plays a pivotal role in the health and disease conditions of which periodontitis is the most highly prevalent disease among the world population [4].

Periodontal infections are a distinct group of clinical entities that are caused by bacterial communities developing in a complex polymicrobial synergistic association in oral microbiome [1]. Approximately 47% of adults in the United States aged between 30 years (approximately 65 million adults) have periodontitis: 30.0% with moderate periodontitis, 8.5% with severe periodontitis, and 8.7% with mild periodontitis. Periodontal infections, in addition to heavy monetary healthcare burden, have also been linked with many systemic diseases [5]. Periodontitis is a chronic inflammatory disease affecting tissues that surround and support the teeth. Its occurrence is associated with important systemic diseases such as cardiovascular disease [6], rheumatoid arthritis [7], and Alzheimer’s disease [8]. One of the most important etiologies of periodontitis is *Porphyromonas gingivalis*, a keystone Gram negative bacterial pathogen [9]. Keystone pathogens can orchestrate inflammatory disease by remodeling a normally benign microbiota, causing an imbalance between normal and pathogenic microbiota (dysbiosis) [9,10,11]. Dysbiosis of oral microbiome in periodontal disease is a hallmark of this condition. Understanding the mechanism of dysbiosis, its functional relevance to disease and strategies to achieve the reversal of dysbiosis to restore health has been the prime focus of research. Recent investigations using the mouse model of this disease have demonstrated that the human periodontal bacterium *Porphyromonas gingivalis* acts as a keystone pathogen in manipulating the normal commensal microbiome into a dysbiotic condition even when present at low abundance; furthermore, this dysbiotic microbiome is causative of disease rather than a consequence of the altered environment in this inflammatory condition [10].

In this article, the oral dysbiosis caused by host-microbiome interaction, and the major mechanisms of dysbiosis and bacterial virulence proteins of co-occurring microbiota that predispose the host to systemic disease are briefly reviewed.

## 2. Oral Microbiota and Microbiome

The term “microbiome” was coined by the Nobel Laureate, Joshua Lederberg. This term was coined to signify the relationship between the micro-organisms and the host such as the symbiotic relationship, namely commensalism, mutualism, and pathogenic. However, these are ignored as determinants of health and disease [12]. Oral microbiome, otherwise known as oral microflora or oral microbiota, is the complex microbial community that resides in the human oral cavity [3]. Approximately 500 to 700 species are estimated to reside in the oral cavity, of which half can be cultivated anaerobically through microbiological techniques, however, half of them still remain uncultivable [13]. Oral microbiome is characterized by cultivation-independent molecular methods using 16s RNA gene based cloning studies [13].

## 3. Oral Microbiome Variations in Health and Disease

The major gateway to the human body is the oral cavity. Micro-organisms enter into the human cavity through food and air, which passes through the nose and then reaches the trachea and lungs through the mouth [3]. Oral microbiome causes a number of oral infectious diseases such as dental caries, periodontitis, endodontic infection, alveolar bone loss, and tonsillitis [3]. Studies have proven that an oral infectious disease will affect the overall health of an individual, extending beyond the oral cavity such as systemic diseases including obstetric convolution [14], cardiovascular disease [15], immunological disorder [16], diabetes [17] and respiratory disease [16,18]. Some of the major systemic implications are dealt with below.

### 3.1. Oral Infection in Pre-Term Birth

Pregnancy gingivitis is a term that indicates inflammation promoted during pregnancy hormonal changes [19] or caused due to the oral consumption of birth control pills [20]. Pre-term birth appears to be a risk factor for neonatal mortality. *Bacteroides forsythus*, *Porphyromonas gingivalis*, *Agregatibacter actinomycetemcomitans*, *Treponema denticola*, *Fusobacterium nucleatum*, *Campylobacter rectus*, *Peptostreptococcus micros*, *Prevotella nigrescens*, and *Prevotella intermedia* are the oral microbiome detected in high levels in mothers of pre-term birth infants [21,22]. The oral infection could mediate the pre-term birth and low birth weight of infants through one or more of the following mechanisms such as (1) the translocation of oral microbiomes to the feto-placental unit through inducing fetal or maternal response that result in the pre-term birth [23,24]; (2) the systemic dissemination of prostaglandins and inflammatory mediators such as IL-6 (Interleukin—6), IL-8 (Interleukin—8), TNF-α (Tumor necrosis factor alpha), and PGE2 (Prostaglandin E2) on the fetal placental unit [24]; and (3) the action of the oral microbiomes reservoir of lipopolysaccharides on the fetal placental unit [24].

Though there is sufficient evidence to prove that periodontal infection remains a significant cause for pre-term birth in infants, it remains conflicted. However, good oral hygiene during pregnancy is one preventive measure.

### 3.2. Oral Infection and Diabetes

Diabetes Mellitus (DM) is a clinical syndrome that is characterized by hyperglycemia, which is caused due to a deficiency of insulin, and affects all age groups. Type 2 diabetes mellitus substantially increases the risk of periodontal disease and has been proposed to modulate oral microbial communities. A shift to a more pathogenic bacterial profile may explain the greater risk of periodontal disease in diabetic patients. Animal studies have provided support for such a shift as a mechanism for the increased risk of periodontitis in diabetes mellitus [25]. Studies have also shown that type 2 diabetes may alter the subgingival bacterial community through changes in substrate availability driven by inflammation and glucose availability [26]. This could be driven by substrate-related alterations in the gingival sulcus in DM that may provide a microenvironment conducive to bacterial growth [27,28,29,30]. Consistent with this, a number of studies have shown that DM alters the colonization of various tissues by pathogenic bacteria. *Helicobacter pylori* infection [31], bacterial infection of foot ulcers, susceptibility to tuberculosis infection, and colonization of the urinary tract by pathogenic bacteria are all significantly enhanced in diabetic individuals [32]. That DM enhances susceptibility to infection by pathogenic bacteria in many different target organs could be due to changes in bacterial substrate availability including sugars and inflammatory products that support bacterial growth. The link between oral infection and diabetes mellitus is yet to be fully recognized and understood by the medical community. However, there are many theories such as (a) chronic hyperglycemia and increased secretion of prostaglandin E_2_ and tumor necrosis factor alpha (TNF-α) are caused by the accumulation of advanced glycation end products; (b) the presence of oral microbiome in the tissue due to impairment of polymorphonuclear leukocyte function [33,34]; and (c) a change in collagen metabolism due to an increase in collagenase activity and decrease in collagen synthesis [35].

Many further studies are currently underway to determine the relationship between oral infection and diabetes in detail [25,26,27,28,29,30,31,32,33,34,35].

### 3.3. Oral Infection and Cardiovascular Diseases

Cardiovascular disease (CVD) is a chronic disease that involves blood vessels or the heart. Cardiovascular disease includes myocardial infarction, atherosclerosis, stroke, and congestive heart failure due to genetic as well as environmental factors [36]. Apart from the above factors, oral microbiome infection also plays a significant role in triggering cardiovascular disease. Among the periodontal bacteria, *Streptococcus sanguinis* and *Porphyromonas gingivalis* are commonly involved in cardiovascular disease [37].

The systemic inflammation of blood vessel walls in the presence of molecular mimicry and inflammatory mediator and inflammation through direct oral microbiome action such as the direct invasion of bacteria to pocket wall and phagocyte-mediated bacterial translocation are the two modes by which the oral microbiome invades the vascular tissue forming acute inflammation, further forming resolution and homeostasis leading to chronic inflammation, which causes cardiovascular disease [38].

## 4. Periodontitis and Associated Bacteria

Periodontal disease is one of the major ubiquitous diseases of the oral microbiome that causes tooth loss in adults [39]. Oral diseases are a disparate group of clinical entities where inflammation results in the loss of attachment between the teeth and gingivae with the formation of periodontal pockets, which later leads to tooth loss contributing to tissue affliction [39,40].

There are two main categories of periodontal disease where the tooth loses its endorsing structures: aggressive periodontitis and chronic periodontitis. These diseases can be characterized beyond the extent of bone loss as generalized or localized, and the austerity of the disease as slight, moderate, or advanced [41]. Most suffer from chronic periodontitis, an insidious disease where the annihilation is consistent with the presence of bacterial plaque and mineralized plaque or calculus [42]. Chronic periodontitis is caused by the variable microbial patterns with polymicrobial infection. In deviance, aggressive periodontitis comprises of rapid attachment loss and bone destruction [43]. A localized form of aggressive periodontitis is an unusually unique disease relative to other forms of periodontitis which usually occurs during adolescence and usually shows a low incidence of periodontal disease; bone resorption in aggressive periodontitis progresses faster than that observed in chronic periodontitis [44], may spontaneously arrest [45], and is localized to distinctive teeth (first molars and incisors); ensuing that the infection tends to aggregate together, indicating that susceptibility to the disease may be genetically regulated [46,47].

Periodontal diseases commence with the aggregation of primary colonizers, usually facultative anaerobes and Gram-positive aerobes such as streptococci, onto the tooth surfaces. This colonization is succeeded by the additional aggregation of late colonizers, which are usually Gram-negative members of the “red complex”, namely *T. denticola*, *T. forsythia*, and *P. gingivalis* in addition to other Gram-negative organisms [48]. The broadly known periodontal pathogens present in plaque are *Porphyromonas gingivalis*, *Treponema denticola*, *Prevotella intermedia*, *Campylobacter rectus*, *Tannerella forsythia*, *Agregatibacter actinomycetemcomitans*, *Selenomonas* spp., *Fusobacterium nucleatum*, *Parvimonas micra*, and *Eubacterium timidum* [49]. Recent microbiome studies have disclosed emerging new pathogens such as *Filifactor alocis*, which may play a significant role in periodontal diseases [50]. There is increasing evidence to suggest that *F. alocis* plays an important role in community dynamics, which thereby could be a major player causing dysbiosis. The unique synergism between *P. gingivalis* and *F. alocis* was also noted in our earlier study [51].

## 5. Yet-Un-Cultivable Bacteria and the Recent Shift in Oral Dysbiosis Research

Recent studies conducted on oral microbiome over the last few years have altered our level of understanding of the polymicrobial communities and their association to health and disease. The increase in the diversity of microbes and their composition gives an idea for the identification and cultivation of more taxon than previously recognized. Some other bacterial species such as *Aggregatibacter actinomycetemcomitans*, *Prevotella intermedia*, *Selenomonas noxia*, *Eubacterium nodatum*, and *Fusobacterium nucleatum* have been found to be correlated with periodontitis, along with the red-complex pathogens [52]. In addition, micro-organisms such as *Desulfobulbus*, *Synergistes*, *Selenomonas*, TM7 (new candidate bacterial division), and *Filifactor alocis* have been identified as potential pathogens [3]. Furthermore, 20% to 60% of the species identified in the oral cavity are yet to be cultivated [3,53].

Recent theories on the etiology of periodontitis have shown a decrease in benign symbioses and an increase in micro-organisms with ameliorated pathogenic potential, favoring a paradigm shift in microbial composition [54]. Hence, an increment in oral microbiome diversity and its composition has led to conclusions that pathogenic communities contain high levels of fastidious and yet-un-cultivated bacteria than previously recognized [3]. Among them, *Filifactor alocis* [55,56] is an emerging pathogen that is present in significantly high numbers in adult periodontitis, refractory periodontitis, endodontic infections [57,58,59], and in aggressive periodontitis [60]. This bacterium has been identified as potential pathogen in a number of independent studies [3,53,61,62,63]. Aruni et al. [51] and Wang et al. [64] have described several of the potential virulence attributes of *F. alocis*. Moreover, Chen et al. [65] recently reported that *F. alocis* was invariably present across various oral habitats in those with periodontitis. *F. alocis* both co-occurred with other pathogens and appeared to play a central role in organizing these pathogens. 

### Filifactor alocis

*F. alocis* is a Gram-positive, asaccharolytic, obligate anaerobic rod that is considered as one of the marker organisms and is an important periodontal pathogen. The organism has now been identified to be significant to the pathogenic structure of biofilms associated with periodontal inflammation [43,62,63]. In comparison with other traditional periodontal pathogens, the high incidence of *F. alocis* in the periodontal pocket when compared with its absence in healthy individuals or those who are periodontitis-resistant has highlighted its importance in the infectious disease process [62,63,66]. This organism, *F. alocis*, has important virulence attributes that can enhance its survival and persistence in the periodontal pocket. One of the most important attributes among them is its relative resistance to oxidative stress; importantly, *F. alocis* has been shown to grow well under such oxidative stress conditions [51]. Furthermore, *F. alocis* has been shown to induce the secretion of many pro-inflammatory cytokines, which in turn could trigger the apoptosis of gingival epithelial cells [67]. It is worth noting that the colonization and survival of *F. alocis* in a mouse model showed a pro-apoptotic local infection that was rapidly resolved by the host neutrophil influx [64]. Moreover, our in vitro studies have shown that *F. alocis* during co-culture with *P. gingivalis* showed an increased invasive capacity of *HeLa* cells [51]. Hence, analysis of emerging research has shown that *F. alocis* is a marker organism for periodontitis. Its unique characteristics that may enhance its virulence potential have been well documented [18,43,51,54,57,64,67,68,69,70,71]. *F. alocis* could be one of the organisms that can play a pivotal role in community dynamics, establishing synergistic partnerships with other pathogenic oral bacteria during the disease state. Hence, it may play a pivotal role in community dynamics orchestrated between the other major players of periodontitis during dysbiotic inflammation. In comparison with other Gram-positive bacteria of the oral cavity, the variations induced in the host proteome during *F. alocis* synergism could lead to many systemic host responses. Therefore, the significance of *F. alocis* putative virulence factors, which may trigger such a key host inflammatory response needs to be explored. It is noteworthy that the incidence of *F. alocis* has been identified in association with both generalized and localized aggressive periodontitis (LAP) in addition to peri-implantitis and endodontic infections. Hence, *F. alocis* is considered an important species of the pathogenic oral microbiome.

## 6. Oral Dysbiosis

Some diseases are caused due to a change in the relationship of microbiome and the host; a decrease in the beneficial symbionts wherein increasing the pathogenic potential causing a microbiome imbalance inside the human body. This process is known as dysbiosis [72]. Oral dysbiosis is the gateway to periodontitis and its associated diseases.

## 7. Causes of Oral Dysbiosis

Oral hygiene is the first and the foremost cause for dysbiosis of oral microbiome. Other major factors causing oral dysbiosis include poor oral hygiene, dietary habits, smoking, gingival inflammation, genetic difference, and dysfunction of the salivary glands such as the activity of salivary proteins [73,74,75]. A dysbiotic shift and the microbiome imbalance in the oral cavity lead to the formation of a biofilm microbial community [76]. According to recent concepts evident through earlier studies, it is known that in a healthy host, pathogens are found to be very few in numbers at healthy sites and hence, oral diseases are caused due to oral microbiome variations rather than exogenous infection [77]. Whereas in dysbiosis, the pathogenic bacteria grow remarkably high with anodyne components on the biofilm surface [77]. This alteration in the formation of biofilm in the oral cavity results in the accumulation of a large proportion of microbes as dental plaque biofilm [78]. Oral dysbiosis not only causes oral related diseases, but also systemic diseases due to the manipulation of a host response.

The subgingival environment provides unique challenges and opportunities for oral bacteria due to the presence of rich immune and inflammatory mediators [79,80,81]. Periodontal health requires a controlled immuno-inflammatory state that can maintain host–microbe homeostasis in the periodontium [82]. However, in periodontitis, the host immune response is dysregulated either due to subversion by the pathogenic oral bacteria or because of defects in host immunoregulation. These processes therefore lead to bacterial outgrowth and facilitates pathogenicity [11]. Hence, as the host immune response is poorly controlled, this generates a self-sustaining pathogenic cycle where dysbiosis and inflammation augment each other. Among the immune cells, neutrophils represent the primary cellular defense in healthy oral tissues. These are the most common leukocytes recruited to the periodontal pocket and are indispensable for periodontal tissue homeostasis. Neutrophils are not adept at phagocytosing biofilm-associated bacteria, which eventually leads to ‘frustrated phagocytosis’ [83]. During this process, neutrophil-derived toxic substances may also be released to the underlying tissue, causing collateral damage to tooth-supportive tissues as they function as double-edged swords, hence collateral damage can be exerted by hyperactive neutrophils or neutrophils in excessive numbers. Many bacteria, especially *P. gingivalis*, can subvert neutrophil functions and related immune responses causing a dysbiotic community through an impaired immune response (Table 1). However, the role of its close synergistic bacterial counterparts (such as *F. alocis*) are yet to be studied in detail.

Specific molecular mechanisms by which periodontal bacteria manipulate the host response to cause dysbiotic inflammation have been elaborately described in other reviews [83,84].

Recently, studies from our lab have shown that the glycan modification due to oral bacterial sialoglycosidases (family of sialic acid modifying enzymes) could play a vital role in the maturation of major virulence factors such as the gingipains and LPS. This in turn could play a role in sialic acid binding ligands in neutrophils leading to neutrophil immune subversion causing dysbiosis (unpublished).

## 8. Oral Bacterial Proteins Involved in Dysbiosis

Certain periodontal pathogens express major virulence proteins that impact apoptotic cell death in gingival epithelial cells (GEC) by disrupting cytokine networks. For example, it has been identified that major virulence proteins of *F. alocis* and *P. gingivalis* modulate epithelial cells on co-infection and are responsible for host cell signaling, metabolic host response, cell-cell interaction, and the regulation of oncogenes in the oral microbiomes [18].

The up regulation of the osteoclast pathway stimulation, increase in alveolar bone resorption, and also tissue degradation through metalloproteinase and inflammatory mediators are induced through the secretion of several cytokines by oral microbiome leading to disrupted cytokine homeostasis and finally tissue degradation [67]. The caspase-3 utilizing extrinsic pathway induces apoptosis and suppresses MEK1/2 expression, which may lead to apoptotic induction [85].

Apoptosis is triggered by the secretion of certain proinflammatory cytokines including tumor necrosis factor alpha (TNF-α), interleukin-1β (IL-1β), and IL-6, which are induced from gingival epithelial cells [67].

Several proteins, namely the microbial surface component-recognizing adhesion matrix molecules (MSCRAMMs), contribute significantly in Gram-positive bacterial virulence by mediating precocious steps in clinical infection such as the adhesion and colonization of host tissues [18]. Since host-pathogen interaction may support the growth and modulation of metabolic processes, the role of amino acid metabolism, especially the arginine most prevalent in the periodontal niche, may be responsible for the overall pathogenicity.

Proteome analysis plays a key role in understanding the process of dysbiotic inflammation through a better understanding of the related molecular mechanisms such as adhesion, invasion, pathogenesis, survival, and adaptation in several oral pathogens such as *Streptococcus oralis* [86], *Streptococcus mutants* [87], *P. gingivalis* [88], and *Fusobacterium nucleatum* [89].

The proteome analysis of *F. alocis* showed several proteases such as the CaaX proteases, metal dependent proteases, calcium dependent proteases and sialoglycoproteases (http://www.ncbi.nlm.nih.gov/genomeprj/46625). The expression of these proteases was found to be elevated in co-culture with *P. gingivalis*. Proteins such as acetyl glutamate kinase, ornithine transaminase, aminotransferase, and glutamate racemase were identified as being involved in ornithine biosynthesis. Several proteins contributing to the virulence potential of *F. alocis* were identified such as the leucotoxin translocation ATP-binding protein, CBARP (voltage dependent calcium channel beta subunit-associated regulatory protein), fibronectin-binding protein, fimbrial assembly protein, Type IV pilus assembly protein, toxin–antitoxin component protein, Hemolysin III type calcium-binding protein, and NAPA (Neutrophil Activating Protein A) [68].

The *F. alocis* proteome contains proteins involved in ornithine biosynthesis and catabolism, urea breakdown such as arginine deiminase, ornithine transaminase, acetyl glutamate kinase, glutamate racemase, amidotransferase, arginine-tRNA ligase, aminotransferase, and arginine decarboxylase [68]. *F. alocis* OTC (ornithine transcarbamylase) has also been shown to be involved in the citrullination of proteins via the ADI (arginine deiminase) pathway. Since protein catabolism is an alternative source of energy in asaccarolytic oral bacteria, heavy amounts of ammonia production occur in periodontal pockets leading to oral dysbiosis [68].

Novel proteins of *P. gingivalis*, namely the sialoglycosidases that interact with sialic acid of the host cells, have recently been considered to play an important role in facilitating dysbiosis [90]. In the saliva rich environment, the mucus containing glycans are considered a vital source as energy for bacterial growth and survival. Manipulation of sialic acid has recently been considered a mechanism to trigger immune evasion through the prevention of sialic acid—siglec (sialic acid binding lectins) interactions in neutrophils. 

Major proteins involved in citrullination have been identified to cause post translational modifications in the host predisposed to rheumatoid arthritis. One of our studies has identified arginine deamininase of *F. alocis* to be involved in a process similar to the peptidyl arginine deiminase of *P. gingivalis* causing peptidyl citrullination [69].

## 9. Proteins of Oral Bacteria Related to Systemic Diseases

Apart from the proteins of oral microbiome that cause oral related disease, there are several other oral bacteria proteins that cause systemic diseases. Proinflammatory mediators such as IL-1β (Interleukin-1β), TNF-α (Tumor necrosis factor—α), and PGE2 (Prostaglandin E2) are released in high levels that exaggerate the host response to the microbes, which causes systemic infection in humans [91,92]. In the case of pre-term birth, elevated levels of cytokine IL-6 have been identified in the host along with IL-1β, TNF-α, and PGE2, which causes infections in the amniotic fluid that lead to complications during delivery [93].

Studies have shown that the protease Gingipain R, released from *P. gingivalis* in large quantities, causes cardiovascular disease by activating factor X, prothrombin, and protein C, thus promoting thrombotic tendency as thrombin is released, the aggregation of subsequent platelets, the transformation of fibrinogen to fibrin, and intravascular clot formation [21]. Furthermore, recent studies have indicated that chronic oral infection induces a high proportion of Hsp65 (Heat Shock Protein) that increases cardiovascular risks [94,95].

Many oral microbiome proteins related to systemic diseases still remain as hypothetical proteins and their functions need to be determined. 

## 10. Stem Cell Modulation by Oral Pathobionts

One of the major effects of pathogenic oral microbiome is manifested in the oral stem cell modulatory processes. Our preliminary study showed secretary proteins of *P. gingivalis* modulated stem cell characteristics. Stem cells contribute to host defense and inflammation. During bacterial-inflammatory disease, stem cells are subjected towards the site of damage, and hence come close to the bacteria and its components. Previous studies using epithelial cells infected by bacteria have shown the modulation of various important functional pathways and changes in gene regulation [51]. Extracellular bacterial proteins secreted in a chronic bacterial infection could modulate stem cells and affect tissue regeneration. Our studies using a transcriptomic approach employing induced pluripotent stem cell iPSCs (induced pluripotent stem cells) were used to evaluate the phenotypic and molecular characteristics. The iPSCs were incubated in different experimental conditions with the secreted proteins of pathogenic bacteria—*Porphyromonas gingivalis*, a commensal bacterium—*Enterococcus faecalis* and a beneficial bacterium—*Lactobacillus casei*—to comparatively evaluate the expression of key genes that govern stemness and differentiation. Secretory proteins of pathogenic bacteria—*P. gingivalis*—were found to possess a modulatory effect both on the stemness and the differentiation of stem cells. It is likely that during a chronic bacterial infection, this could prolong stemness and prevent differentiation, hence, sustaining the infectious state and preventing cell recovery.

## 11. Animal Models for Studying Systemic Diseases Caused Due to Periodontitis

Experimentally induced animal models are critically important and are used to study and analyze different aspects of oral disease as well as the systemic diseases caused due to oral dysbiosis. Though human cell culture or in vitro cell culture have been found to be useful models, the findings regarding the host-microbe interaction were anonymous and obscure, hence why the animal model (in vivo) came into limelight [96]. A detailed review on this topic can be found in [96].

Nonhuman primates have similar oral structures to that of humans. In particular, rhesus monkeys (*Macaca mulatta*), cynomolgus monkeys (*Macaca fascicularis*), and baboons (*Papioanubis*) are susceptible to disease. They have been inoculated with human pathogens and subsequently tested. However, they are prone to tuberculosis, which makes them a less practical model [97].

Minnesota miniature pigs have maxillofacial and oral structures analogous to those of humans in terms of physiology, anatomy, and disease progression [98]. However, miniatures pigs are relatively expensive and only very few studies are available to support their use [96].

The host-microbiome interactions in the rodent model are similar to humans. Rats are often used as an effective model. They are inexpensive and obtained easily with different microbial status. Sometimes swamp rice rats (*Oryzomys palustris*) are widely used to assess some therapeutic models and dietary effects [96,98].

The Baker mouse model, chemically induced model, and murine back abscess model have been used to examine the alveolar bone resorption, inflammatory response, and the interaction of host response and oral microbiome to various phylotypes that leads to systemic disease [99], respectively. However, since they are small in size, they are needed in large quantities.

Rabbits, ferrets, and hamsters have also been used as animal models. Each of the animal models has its own advantages and disadvantages, though most of them show similarities to human disease. Among these, mice and rat models are useful for understanding certain aspects of host-microbiome interaction and their therapies, and have been successfully used to study the “dysiosis-rebiosis” concept of oral health restoration.

Though the animal models provide a huge number of findings and data, it is occasionally arduous to ascertain whether the findings and data are pertinent to humans [96].

## 12. Conclusions

Recent studies have paved a paradigm shift in emphasizing the oral ecosystem as vital to maintaining both the oral and overall health of the body. Maintaining microbial equilibrium within oral cavity protects pathogens from manifesting a disease state. Disturbing the homeostasis of the oral cavity can flare pathogen activity leading to a disease state. As the oral cavity is the primary gateway to the body, this may result in spreading infection to other body sites producing systemic diseases. What is known up until now is only about the oral bacterial species that can be cultivated and sequenced and obtained from pure culture approaches. However, this does not reflect their actual behavior in complex microbial communities as the species that have been identified by culture independent methods are still classified as uncultivated phylotypes. Their impact on community dynamics and their role in oral dysbiosis are yet to be fully explored. Furthermore, newer concepts to study dysbiosis through immune subversion mechanisms involving gycan mediated pathogen-host interactions are currently being explored. Recent research focusing on bacteria such as *F. alocis* and other new candidates are gaining momentum. Their collective role in community dynamics is yet to be clearly explored. Hence, an insight into community level physiological and metabolic capacity will help find new cures to modulate the activity of such communities and steer them towards a health state. Their synergistic and competitive contributions to oral dysbiosis could reveal new insight into specific species, or its genes or pathways of interest to develop novel therapeutics. Analysis of human oral microbiome will significantly contribute to the development of precise medical interventions.

## Figures and Tables

**Table 1:** Table showing various mechanisms of immune subversion and their outcome [84].

Mechanisms	Outcome
Whole cells, LPS bind to adhesion molecules (IL-8, ICAM-1, E-selectin).	Impaired recruitment
SerB suppression of IL-8 production by dephosphorylation of the Ser536 of NF-kB p65 preventing nuclear translocation and transcription.	IL-8 production suppressed
Bacterial binding to FMLP and PPAD-citrullinated C5a.	Reduced chemotaxis
Dual regulation of TREM-1 by Arg- and Lys-gingipain. Outcome depends on infection stage.	Evasion of host defense
Resistance to killing by granular contents. C5 convertase-like activity produces C5a, which is involved in subversion of C5aR TLR2 crosstalk. This leads to My88D degradation, PI3K activation and inhibition of RhoA GTPase.	Killing prevented. Inhibits antimicrobial response and promotes inflammatory response
Activated CR3 interacts with P. gingivalis fimbriae and induces downregulation of IL-12p70 a key cytokine in intracellular bacterial clearance.	Reduced bacterial clearance
LPS and lipid A delay neutrophil apoptosis through TLR2 signaling	Prolonged acute inflammation
